# Testing Is More Desirable When It Is Adaptive and Still Desirable When Compared to Note-Taking

**DOI:** 10.3389/fpsyg.2018.02596

**Published:** 2018-12-18

**Authors:** Svenja Heitmann, Axel Grund, Kirsten Berthold, Stefan Fries, Julian Roelle

**Affiliations:** ^1^Department of Educational Sciences, Ruhr-Universität Bochum, Bochum, Germany; ^2^Department of Psychology, Bielefeld University, Bielefeld, Germany

**Keywords:** testing, test-based learning, desirable difficulties, adaptivity, note-taking, focusing

## Abstract

Testing is a well-established desirable difficulty. Yet there are still some open issues regarding the benefits of testing that need to be addressed. First, the possibility to increase its benefits by adapting the sequence of test questions to the learners’ level of knowledge has scarcely been explored. In view of theories that emphasize the benefits of adapting learning tasks to learner knowledge, it is reasonable to assume that the common practice of providing all learners with the same test questions is not optimal. Second, it is an open question as to whether the testing effect prevails if stronger control conditions than the typical restudy condition are used. We addressed these issues in an experiment with *N* = 200 university students who were randomly assigned to (a) adaptive testing, (b) non-adaptive testing, or note-taking (c) without or (d) with focus guidance. In an initial study phase, all participants watched an e-lecture. Afterward, they processed its content according to their assigned conditions. One week later, all learners took a posttest. As main results, we found that adaptive testing yielded higher learning outcomes than non-adaptive testing. These benefits were mediated by the adaptive learners’ higher testing performance and lower perceived cognitive demand during testing. Furthermore, we found that both testing groups outperformed the note-taking groups. Jointly, our results show that the benefits of testing can be enhanced by adapting the sequence of test questions to learners’ knowledge and that testing can be more effective than note-taking.

## Introduction

Testing is a well-established desirable difficulty. Making the learning process more difficult by providing learners with test questions that require retrieval from memory after an initial study phase has been shown to foster retention and transfer (Roediger and Butler, 2011; Dunlosky et al., 2013; Rowland, 2014; Adesope et al., 2017; Pan and Rickard, 2018). However, even though the effects reported in the literature are robust, there are still some open issues regarding the benefits of testing that should be addressed.

First, it is unclear whether the full potential of testing has yet been exploited. In real educational settings such as regular high-school lessons or university lectures, testing is often implemented by having all learners answer the same pre-set sequence of specific test questions (e.g., Mayer et al., 2009; McDaniel et al., 2011; McDermott et al., 2014). However, a wealth of research clearly highlights that the benefits of learning tasks substantially depend on the fit between the learners’ level of knowledge and the respective learning task (e.g., Metcalfe, 2002; Kalyuga, 2007; Kalyuga and Renkl, 2010; Roelle et al., 2015a). It follows that testing in its usual form might be suboptimal, and that it would be more effective to provide learners with test questions that are adapted to their level of knowledge. The primary purpose of the present study was to test this hypothesis.

Second, a recent discussion regarding which control condition the benefits of testing should be compared to has gained momentum. Specifically, it has been argued that the commonly used restudy control condition might be too weak because restudy has relatively low utility as a learning method (Dunlosky et al., 2013). Comparing testing against restudy could therefore lead to an overestimation of the testing effect (McDaniel et al., 2009; Kornell et al., 2012). In response, note-taking has been suggested as a stronger alternative (McDaniel et al., 2009; Rummer et al., 2017; Miyatsu et al., 2018). Yet empirical findings that illuminate the effects between testing and note-taking are scarce. Thus, the secondary purpose of this study was to contribute to the analysis of the impact that note-taking as a control condition has on the standing of the testing effect.

To address these issues, we conducted an experiment in which all learners watched an e-lecture in an initial study phase. As a follow-up learning task, the learners received either an adaptive or a non-adaptive sequence of specific test questions (*testing conditions*) or were assigned to one out of two *note-taking conditions*. The note-taking conditions differed in whether learners were guided to focus on the same learning content items as the learners who received test questions. Learning outcomes measured after 1 week delay served as the main dependent variable.

### The Benefits of Adaptive Testing

Testing requires learners to actively retrieve information from memory and is therefore more difficult than other learning activities that require little to no retrieval such as restudying and note-taking. However, although it is more difficult, this form of retrieval has beneficial effects on learning outcomes. Research clearly shows that learners substantially benefit from various forms of retrieval practice (e.g., Rawson and Dunlosky, 2012; Blunt and Karpicke, 2014; Lechuga et al., 2015; Bae et al., 2018; Eglington and Kang, 2018; for recent overviews see Roediger and Butler, 2011; Karpicke and Grimaldi, 2012; Rowland, 2014; Adesope et al., 2017), thus making the difficulty induced via testing desirable (Bjork and Bjork, 1992; Metcalfe, 2011).

There are two basic ways to implement testing with desirable effects: (a) the provision of free recall prompts (e.g., Roediger and Karpicke, 2006; Brewer et al., 2010; Zaromb and Roediger, 2010; Lipowski et al., 2014; Dobson and Linderholm, 2015; Rummer et al., 2017) or (b) the provision of specific test questions (e.g., Butler et al., 2007; Kang et al., 2007; Carpenter et al., 2008; Meyer and Logan, 2013; Roelle et al., 2018). The main difference between these two types of testing lies in the extent to which learners are guided to focus on specific (relevant) content items. In free recall, learners usually do not receive any focus guidance and thus recall all of the learning content freely (*general testing*). By contrast, in *specific testing*, learners reply to specific test questions that are directed at certain but not necessarily all learning content items.

In view of the fact that in real educational settings, such as high-school lessons or university lectures, the desirable difficulty of testing is frequently implemented via the provision of specific test questions (also referred to as *practice quizzing*, e.g., Mayer et al., 2009; McDaniel et al., 2011; Jensen et al., 2014; McDermott et al., 2014), in the present study we focused on the specific testing approach. Similar to general testing, several studies show that specific testing entails beneficial effects (Adesope et al., 2017; Rowland, 2014). Nevertheless, it is reasonable to assume that it has not yet reached its full potential because the extent to which learners are provided with the *right* test questions has received scarce attention so far.

Generally, the research on specific testing indicates that providing learners with *higher-level* test questions that require not only retrieval but also the comprehension and application of certain content items can be more effective than *lower-level* test questions that merely require retrieval (e.g., Jiang and Elen, 2011; Jensen et al., 2014; Roelle et al., 2018). On this basis, it could be proposed that in specific testing, providing learners with mostly higher-level test questions would be especially beneficial. In a possible implementation that is based on this notion, learners would receive only a few lower-level test questions that merely require the retrieval of basic content items at the beginning and would then quickly proceed to more complex higher-level test questions that also require comprehension and application.

However, providing learners with a pre-set sequence of specific test questions that includes mostly higher-level test questions may not come without costs. Higher-level test questions usually require that learners retrieve and simultaneously process a higher number of content items and thus are more *complex* than lower-level test questions. This, in turn, makes testing more difficult, which can create undesirable consequences. There is evidence that if testing is too difficult, it can become ineffective or even counter-effective (e.g., Karpicke et al., 2014; van Gog and Sweller, 2015; Carpenter et al., 2016; Roelle and Berthold, 2017). In this case, testing does not induce desirable but undesirable difficulties. Thus, it can be predicted that in order to optimize specific testing, establishing a close fit between the learners’ level of knowledge and the test questions could be more beneficial than increasing the complexity of the test questions provided without taking learner knowledge into account.

This prediction is in line with both the *expertise reversal framework* (e.g., Kalyuga, 2007; Kalyuga et al., 2012; Kalyuga and Singh, 2016) and the *region of proximal learning framework* (e.g., Metcalfe, 2002, 2009). The expertise reversal framework theorizes that instruction that is suitable for novices may not be suitable for more knowledgeable learners and vice versa because both redundant processing (too easy lower-level learning tasks) and processing that cognitively overloads learners (too complex higher-level learning tasks) inhibits learning (Chen et al., 2016). Similarly, the region of proximal learning framework assumes that learners benefit more from learning tasks whose complexity is just one step further from the content that has already been learned. Hence, both frameworks predict that learner knowledge and learning task complexity should be matched in order to exploit the full potential of the learning arrangement. The theoretical notion that learning task complexity should be adapted to the learners’ level of knowledge is furthermore supported by several empirical findings. It has, for instance, been shown that adaptivity enhances the benefits of completion tasks (Corbalan et al., 2009), a combination of worked examples and problem solving (Kalyuga and Sweller, 2005), practical training tasks (Salden et al., 2004), and feedback (Roelle et al., 2015b).

In the present study we compared a non-adaptive implementation of specific testing that proceeded from simple to complex higher-level test questions without taking learners’ knowledge into account (*non-adaptive testing*) to an adaptive approach in which test question complexity was determined by the learner’s knowledge (*adaptive testing*). The learners’ level of knowledge was diagnosed by how they answered a previous test question that had covered the same content.

Such an adaptive testing approach could foster learning in comparison to non-adaptive testing via two mediators. First, adaptive testing could substantially enhance performance on the test questions in the learning phase. At least in the case that non-adaptive testing increases test question complexity too quickly, learners who receive adapted test questions should outperform their counterparts in terms of testing performance. As testing performance has been shown to be an important mediator of the benefits of testing (Rowland, 2014), it follows that adaptive testing could foster learning outcomes in comparison to non-adaptive testing via increased testing performance. Second, adaptive testing could reduce cognitive demand by circumventing test questions that are too complex. Mediation analyses have shown that excessive cognitive demand can detrimentally affect learning outcomes in various learning settings (e.g., Roelle et al., 2015a; Glogger-Frey et al., 2017). Additionally, test questions that place a high cognitive demand could lead to cognitive exhaustion over time (Schnotz, 2010), thus leading to a further reduction of learning outcomes. Hence, in comparison to non-adaptive testing, adaptive testing could foster learning outcomes not only via higher testing performance but also via lower cognitive demand in the learning phase.

### Testing: Compared to What?

Testing is often evaluated by pitching its results against a restudy control condition (e.g., Roediger and Karpicke, 2006; Agarwal et al., 2008; Carpenter et al., 2008; Johnson and Mayer, 2009; Meyer and Logan, 2013; Lipowski et al., 2014; Endres and Renkl, 2015; for an overview, see van Gog and Sweller, 2015). Restudy control conditions aim at exposing the learners to the learning material in the same way they experience it during the initial learning phase (e.g., by asking the learners to reread a text). However, as Kornell et al. (2012) have pointed out, using restudy as a control condition might lead to an overestimation of the testing effect. Restudy seems to be of low utility for learning and generally yields lower learning outcomes than other, more effective techniques (Callender and McDaniel, 2009; Dunlosky et al., 2013). One could argue that a control condition of at least moderate utility should be employed to appropriately evaluate testing in terms of its educational utility.

Note-taking could be a suitable alternative to restudy because it is a commonly used and effective strategy (McDaniel et al., 2009; Rummer et al., 2017; Miyatsu et al., 2018). When used as a learning method, learners write down notes on the learning material in their own words. Note-taking presumably requires small doses of retrieval; learners might, for example, add aurally presented information to a handout or presentation slides after a lecture as part of their learning routine. As note-taking requires considerably more cognitive effort than restudy, one can assume that it can function as a stronger control condition than restudy. Rummer et al. (2017) recently conducted a study in which they compared free recall testing (i.e., general testing) with a note-taking control group. They reported that the testing effect did not prevail after 1 week. This finding shows that replacing restudy control conditions with note-taking could indeed result in a stricter and therefore more educationally relevant evaluation of the utility of testing and the testing effect. However, in terms of establishing the benefits of *specific* testing, even a note-taking condition might not be a suitable control condition. Contrary to general testing via free recall, specific testing does not only engage learners in retrieval, but also focuses their attention on specific content items, which can further enhance learning outcomes. The literature on *relevance instructions* clearly indicates that focusing the processing onto relevant main content items can have a beneficial effect on learning outcomes (e.g., Berthold and Renkl, 2010; Renkl, 2015; Roelle et al., 2015a; for an overview, see McCrudden and Schraw, 2007). Instructions on note-taking, on the other hand, usually leave it up to the learner to decide on which content to focus. In comparison to specific testing, this should not only lead to a higher diffusion of attention across all content items, but also to less processing of the content that, from the perspective of later criterion tests, is most relevant. In terms of learning outcomes, it can therefore be argued that specific testing provides an advantage over note-taking beyond the engagement in testing, which could undermine the strength of note-taking as a control group. If learners who engaged in note-taking would receive focusing guidance as well, this advantage would be canceled out, increasing the condition’s suitability as a control condition. In the present study, we therefore included not only a regular note-taking condition but also a note-taking condition in which the learners’ attention was guided toward focusing on the same content items at which the test questions were directed.

### Research Questions

Based on our theoretical considerations, the primary purpose of our study was to investigate the potential advantages of an adaptive approach to specific testing. Specifically, we were interested in whether adaptive testing would yield higher learning outcomes than non-adaptive testing (research question 1) and if the effects between adaptive and non-adaptive testing would be driven by differences between the conditions concerning (a) the performance on the test questions and (b) the perceived cognitive demand during testing (research question 2). A secondary goal of our study was to contribute to the research that analyzes the benefits of testing in comparison to stronger control conditions than restudy. Specifically, we wanted to know whether testing that is implemented via the provision of specific test questions would be more beneficial than both note-taking and focused note-taking (research question 3).

## Materials and Methods

### Sample and Design

In light of the notions that the potential benefits of (a) adaptive testing in comparison to non-adaptive testing and (b) testing in comparison to (focused) note-taking would be educationally relevant if they would be of at least medium size (*f* = 0.25; $\eta_{p}^{2}$ = 0.06; *d* = 0.50), we conducted an a priori power analysis using G^∗^Power 3.1.9.2 (Faul et al., 2007) to determine the sample size needed to establish a power of at least 80 %. The power calculation yielded a required sample size of *N* = 180. Against this background, we recruited *N* = 200 university students as participants for our experiment (137 female; *M_Age_* = 24.29, *SD* = 4.87). Due to non-compliance, *n* = 13 participants had to be excluded from analysis, resulting in a final sample size of *N* = 187. The learners were randomly assigned to one condition of a one factorial between-subjects design that comprised four conditions. In an initial study phase, all learners watched an e-lecture. The experimental manipulation took place in the learning phase that occurred after the initial study phase. In this learning phase (80 min), the participants learned either by (a) *adaptive testing*, (b) *non-adaptive testing*, (c) *note-taking*, or (d) *focused note-taking*. The learners received €40 for their participation. The experiment was approved by the Ethics Committee of Bielefeld University, Germany (No. 2017 – 049) and written informed consent was obtained from all participants.

### Materials

#### E-lecture

In an initial study phase, all participants watched an e-lecture on the topic of social norms that comprised 26 narrated slides and covered the norm of reciprocity, the norm of commitment, and the norm of obedience. For the subtopic of reciprocity, the participants learned about the definition of the norm, D. T. Regan’s seminal study on reciprocity, and the mechanisms and conditions of the door-in-the-face technique. The part about the norm of commitment covered the definition of the norm, Moriaty’s *Beach Blanket* study, and the mechanisms and conditions of the low-ball technique. The last part of the e-lecture centered on the norm of obedience. It covered its definition and the factors that make obedience more likely as well as Milgram’s study on obedience. The e-lecture lasted approximately 30 min.

#### Testing

For the two testing conditions, specific test questions spanning across four levels of complexity were created. The test questions were explicitly designed not to cover the entirety of the content presented in the e-lecture, but to leave some content untested. Level 1 (low-level) test questions required the learners to reproduce main content from the e-lecture one subtopic at a time. Depending on the social norm, this was either the definition of the norm or a technique that was based on it, and their respective conditions (e.g., “Describe the procedure of the door-in-the-face technique. Please also list the conditions relevant for the technique’s success and the resulting outcome of those conditions.”).

In the more complex higher-level test questions (Levels 2, 3, and 4), the learners were asked to analyze a situation described at the beginning of the test question in which the respective social norms or techniques were applied. The Level 2 test questions asked for the analysis of the situation with respect to only *one* social norm or technique (e.g., “Anja starts her first job as a teacher at a high school. At first, her senior students think she is a student, too. Anja cannot blame them; after all, she is only 5′3,” looks very young, and dresses casually. Analyze the situation by taking into account your knowledge of the six factors which foster obedience: (a) Which of those factors are met regarding Anja and her senior students, (b) which factors are not met, and (c) for which factors is it not possible to reach a conclusion from the information provided in the text? Justify your answers by providing short explanations.”). On Level 3, the test questions required learners to simultaneously consider *two* social norms or techniques at a time (e.g., reciprocity × commitment); in the Level 4 test questions, all three social norms or techniques were intertwined (reciprocity × commitment × obedience). In both testing conditions, the subtopics alternated between Levels 1, 2, and 3, so that the same subtopic(s) would not be presented back to back.

**FIGURE 1:**
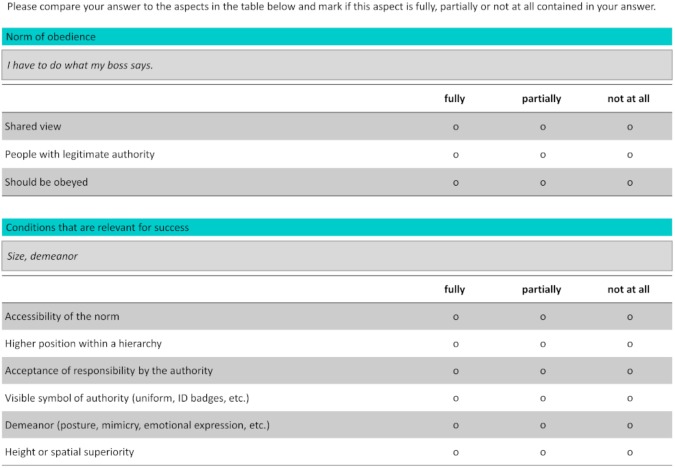
Feedback mask as used during the learning phase of the testing conditions (translated from German).

After each test question, the participants evaluated their responses by using a feedback form (see Figure 1). For each part of a test question, the feedback form provided the answer given by the learner and the correct answer, which was broken down into idea units. The idea units were obtained by analyzing the correct answer and extracting the single statements needed to construct the complete meaning of the correct answer (e.g., *The shared view that those with legitimate authority should be obeyed* contains the idea units *shared view, people with legitimate authority*, and *should be obeyed*). The participants then had to score their answers by evaluating if the idea units were *fully, partially*, or *not at all* included in their answer. This procedure was based on the feedback method used in a study by Zamary et al. (2016). Providing feedback broken down into idea units helps learners to accurately self-evaluate their answers (Lipko et al., 2009; Dunlosky et al., 2011). The learners received examples and detailed instructions on how to score their answers before starting the learning phase.

Once the participants had evaluated their answers, a score was automatically computed for the test question. This score provided the basis for the adaption in the adaptive testing group. Proportion scores were calculated using the theoretical maximum score for each test question. Proportion scores ≤ 0.50 meant the next test question would be one of a lower level, scores ≥ 0.85 meant the participant would receive a test question of a higher-level. The participants remained on the current test question level if their score was >0.50 but <0.85. On Levels 1 and 4, it was not possible to receive a lower- or a higher-level test question. In order to avoid having the participants repeatedly answer the same test questions, we created parallel versions of the test questions on Levels 2, 3, and 4. The parallel test questions required similar content items and asked the same open-ended questions but were based on different situations.

In the non-adaptive testing condition, the participants received a pre-set sequence of test questions of increasing complexity. This sequence followed a simple-to-complex logic. Specifically, it contained three questions on Levels 1–3, meaning the participants first received three Level 1 questions (reciprocity, commitment, and obedience) and then three Level 2 questions (reciprocity, commitment, and obedience), followed by three Level 3 questions (reciprocity × commitment; commitment × obedience; obedience × reciprocity). When they reached the final level, the participants then received Level 4 test questions for the remainder of the learning phase.

#### Note-Taking

**FIGURE 2:**
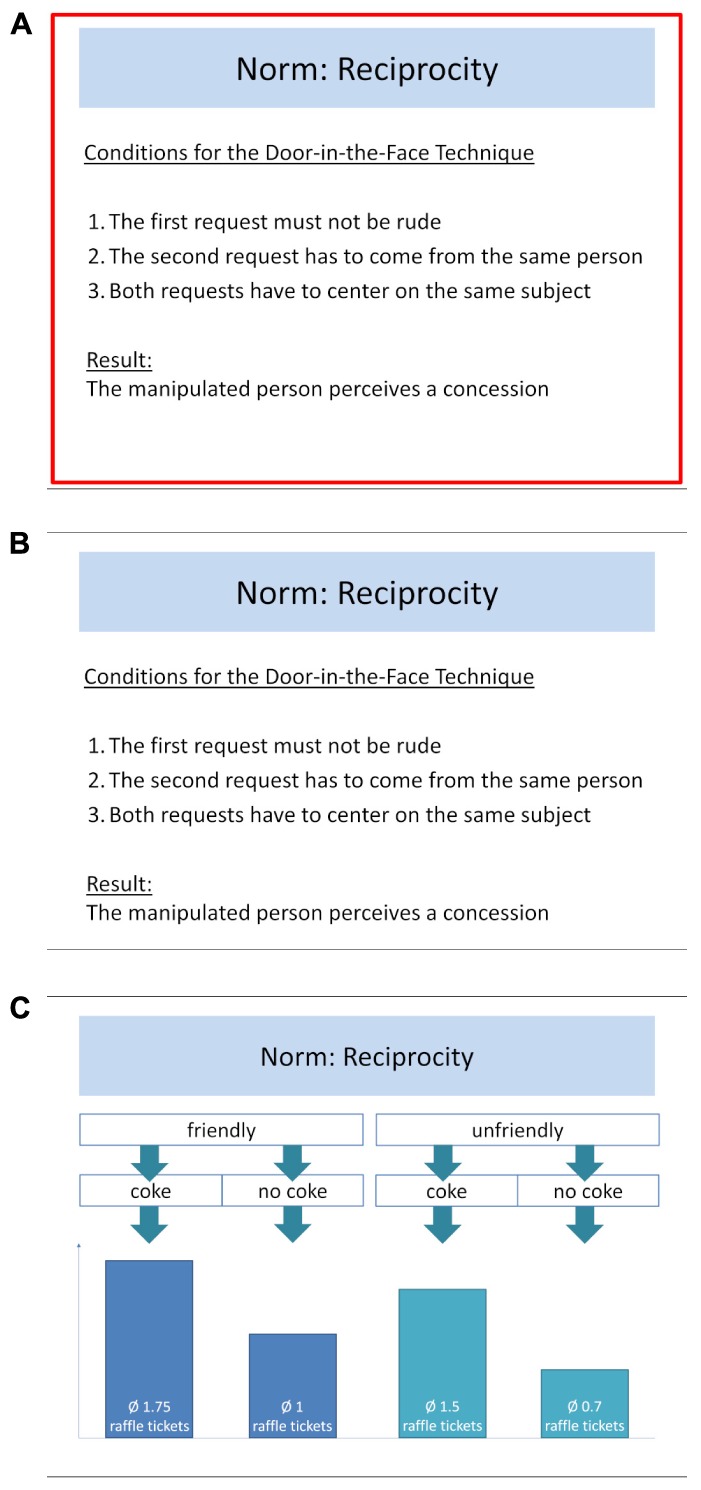
Sample slides as used in the e-lecture during the initial study phase and during the learning phase of the note-taking conditions (translated from German). **(A)** Slide showing tested content as seen by the focused note-taking group (with a frame). **(B)** The same slide as seen by the note-taking group (without a frame). **(C)** Slide showing untested content as seen in the learning phase of both the note-taking and focused note-taking conditions.

The note-taking conditions received the slides that were used for the e-lecture as their learning material. Participants could navigate freely between the slides and were instructed to write down notes in text-boxes that were located next to them. There was no time limit on viewing each slide. For the focused note-taking group, some of the slides were framed in bright red. The content on these slides was also covered by the test questions (*tested content*; see Section “Testing”). The learners were asked to focus their learning efforts on these slides (“During your study, please put special emphasis on the slides with a red frame.”). Content on slides that did not include red frames (*untested content*, i.e., content that was not covered by the test questions) was semantically associated with the focused tested content but was not crucial for its understanding. Untested content could therefore potentially serve as a retrieval cue for the respective tested content. For instance, untested content slides provided a definition of the norm (which helped to understand the mechanics of a technique), a study that illustrated a norm, and/or provided information on the relevance of the norm in everyday life or for historic events (see Figure 2 for example slides). The testing groups should therefore not be at a disadvantage for understanding tested content by not receiving untested content during their learning phase.

### Instruments and Measures

#### Pre- and Posttest

We created a pretest to measure the participants’ prior knowledge on the topic of social norms before the initial learning phase and a posttest to measure learning outcomes thereafter. Both tests contained the same 22 questions. Thirteen of the questions covered the content at which the test questions in the learning phase had been focused (subscale *tested content*, posttest Cronbach’s α = 0.82). The remaining nine questions tested the participants’ knowledge regarding content at which the test questions had not been directed (subscale *untested content*, posttest Cronbach’s α = 0.70). The questions on the tested content can be further differentiated into the subscales *familiar questions, near transfer questions*, and *generation questions*. The familiar questions were questions that the participants of the testing conditions had already answered on Level 1 in the learning phase (posttest Cronbach’s α = 0.68). The near transfer questions were structurally similar to the Level 2, 3, and 4 test questions, but related to different situations than the test questions (posttest Cronbach’s α = 0.76). The generation questions asked the learners to generate their own example situations that illustrated the social norm or technique. There was one generation question for each of the three social norms or techniques (posttest Cronbach’s α = 0.62).

All questions were scored by independent raters using a scoring protocol that contained idea units of the correct answers for each question. Interrater reliability, as determined by the intraclass coefficient with measures of absolute agreement, was very high for each of the questions (all ICCs > 0.85). The final scores were obtained by calculating proportion scores of the theoretical maximum score for each question. The proportion scores were then aggregated to determine the scores regarding the aforementioned subscales tested content, familiar questions, near transfer questions, generation questions, untested content as well as the subscores for the four question levels by averaging the respective question scores. By using this procedure, each question was given the same weight (theoretical min: 0; theoretical max: 1).

#### Test Question Performance

Performance on the test questions was obtained by using the participants’ self-scores (see Testing) and calculating proportion scores of the theoretical maximum score for each test question. These proportion scores were then averaged for each test question level which led to all test questions having equal weight (theoretical min: 0; theoretical max: 1).

#### Cognitive Demand of Test Questions

In the testing conditions, the perceived cognitive demand was assessed for every test question before the learners received the feedback. For this purpose, we asked the learners, “How demanding did you find the question?” using an 11-point Likert scale ranked from (1) v*ery low degree of cognitive demand* to (11) *very high degree of cognitive demand*. These scores were then averaged for each test question level. As this measure was linked to the test questions, it was only restricted to the testing conditions.

### Procedure

In all conditions, the participants worked individually in a digital learning environment on computers in a laboratory. First, all learners took the pretest. After finishing the pretest, they were asked to watch the e-lecture attentively because they would be given questions on its content afterward (initial learning phase). Following the e-lecture, the participants had to take a 5-min break before the experiment resumed. Afterward, they received instructions for the subsequent learning phase according to their condition and processed the e-lecture content accordingly. The learning phase had a duration of 80 min in all conditions. At the end, the participants were informed that the experiment would continue in one week’s time and were asked not to deal with the subject of social norms until then. In the second experimental session, the participants took the posttest^1^.

## Results

An α-level of 0.05 was used for all statistical analyses (we report both two-tailed and one-tailed *p*-values for all analyses). For *F* tests, we report partial η^2^ as the effect size measure. In line with Cohen (1988), we consider values of approximately 0.01 as small, of approximately 0.06 as medium, and of approximately 0.14 or more as large effect sizes. As effect size measure for *t-*tests, we report Cohen’s *d* qualifying values of approximately 0.20 as small effects, values of approximately 0.50 as medium effects and values of 0.80 as large effects.

### Preliminary Analyses

To test whether the random assignment resulted in comparable groups, we compared the learners’ pretest scores and grade point average. Overall, pretest scores were very low (*M* = 4.1% of the maximum attainable score). Surprisingly, there was a statistically significant difference between the groups regarding prior knowledge, *F*(3,183) = 11.79, *p* < 0.001, $\eta_{p}^{2}$ = 0.16. The effect was due to the fact that the note-taking groups (5.1 and 4.9%) scored higher than the testing groups (3.0 and 3.2%). Although it is likely that these differences were of little practical relevance, we included the pretest score as a covariate in our subsequent analyses regarding learning outcomes. There was no statistically significant difference between the groups in terms of grade point average, *F*(3,183) = 0.38, *p* = 0.767, $\eta_{p}^{2}$ = 0.01.

### Adaptive vs. Non-adaptive Testing

#### Learning Outcomes

In research question 1, we were interested in whether adaptive testing would yield higher learning outcomes than non-adaptive testing. An ANCOVA revealed a statistically significant effect on the overall posttest score, with the adaptive group scoring higher than the non-adaptive group, *F*(1,89) = 7.67, *p* = 0.007, $\eta_{p}^{2}$ = 0.08 (one tailed: *p* = 0.004). To enable a more differentiated interpretation of the results, we also analyzed the subscales of the posttest (see Table 1 for pretest and posttest scores). The adaptive testing group performed better on the tested content questions, *F*(1,89) = 10.36, *p* = 0.002, $\eta_{p}^{2}$ = 0.10 (one-tailed: *p* = 0.001) but not on untested content questions, *F*(1,89) = 0.56, *p* = 0.457, $\eta_{p}^{2}$ = 0.01 (one-tailed: *p* = 0.229). Furthermore, within the tested content questions, the adaptive group outperformed the non-adaptive group regarding performance on familiar questions, *F*(1,89) = 13.82, *p* < 0.001, $\eta_{p}^{2}$ = 0.13 (one-tailed: *p* < 0.001), near transfer, *F*(1,89) = 5.65, *p* = 0.020, $\eta_{p}^{2}$ = 0.06 (one-tailed: *p* = 0.010), and generation questions, *F*(1,89) = 3.14, *p* = 0.080, $\eta_{p}^{2}$ = 0.03 (one-tailed: *p* = 0.040). The tested content questions were also differentiated with respect to question level. The adaptive group performed better on Levels 1 and 2 posttest questions, *F*(1,89) = 13.82, *p* < 0.001, $\eta_{p}^{2}$ = 0.13 (one-tailed: *p* < 0.001), and *F*(1,89) = 18.59, *p* < 0.001, $\eta_{p}^{2}$ = 0.17 (one-tailed: *p* < 0.001). No significant effects were found for Levels 3 and 4, *F*(1,89) = 0.19, *p* = 0.667, $\eta_{p}^{2}$ = 0.00 (one-tailed: *p* = 0.334), and *F*(1,89) = 0.03, *p* = 0.862, $\eta_{p}^{2}$ = 0.00 (one-tailed: *p* = 0.431).

**Table 1:** Means and standard deviations for pre- and posttest scores by scales for the adaptive testing group, non-adaptive testing group, note-taking-group, and focused note-taking group.

		Adaptive testing *n* = 43				Non-adaptive testing *n* = 49				Note-taking *n* = 46				Focused note-taking *n* = 49			
					
		Pretest		Posttest		Pretest		Posttest		Pretest		Posttest		Pretest		Posttest	
									
Scale	Q	*M*	*SD*	*M*	*SD*	*M*	*SD*	*M*	*SD*	*M*	*SD*	*M*	*SD*	*M*	*SD*	*M*	*SD*
Overall	22	0.03	0.02	0.41	0.11	0.03	0.02	0.35	0.11	0.05	0.02	0.38	0.11	0.05	0.03	0.37	0.12
Tested content	13	0.03	0.01	0.53	0.12	0.03	0.02	0.45	0.12	0.04	0.03	0.37	0.11	0.04	0.03	0.38	0.11
Untested content	9	0.03	0.04	0.23	0.14	0.03	0.04	0.21	0.14	0.07	0.04	0.38	0.14	0.06	0.05	0.36	0.16
Familiar questions	3	0.00	0.01	0.54	0.15	0.00	0.01	0.42	0.17	0.00	0.01	0.41	0.17	0.00	0.00	0.37	0.17
Near transfer	7	0.05	0.02	0.47	0.14	0.06	0.04	0.40	0.12	0.07	0.05	0.28	0.10	0.07	0.05	0.30	0.11
Generation	3	0.01	0.03	0.66	0.17	0.01	0.02	0.59	0.22	0.01	0.03	0.53	0.22	0.02	0.03	0.57	0.16
Level 1	3	0.00	0.01	0.54	0.15	0.00	0.01	0.42	0.17	0.00	0.01	0.41	0.17	0.00	0.00	0.37	0.17
Level 2	6	0.06	0.02	0.55	0.13	0.06	0.02	0.43	0.13	0.06	0.03	0.40	0.12	0.07	0.03	0.43	0.10
Level 3	3	0.01	0.03	0.54	0.19	0.02	0.06	0.55	0.21	0.03	0.07	0.34	0.18	0.03	0.08	0.34	0.17
Level 4	1	0.00	0.02	0.36	0.25	0.01	0.05	0.35	0.19	0.02	0.05	0.15	0.17	0.01	0.05	0.20	0.17


*Q, number of questions taken into account for this score.*

#### Learning Processes

In research question 2, we were interested in whether the potential effects between adaptive and non-adaptive testing would be driven by differences between the conditions concerning performance on the test questions and the cognitive demand of the test questions in the test-based learning phase. To address this research question, in the first step we used *t*-tests to analyze the extent to which the groups differed on these learning process measures (Table 2 provides an overview of the descriptive measures).

**Table 2:** *n*, means, standard deviations, and effect sizes *d* for the number of test questions answered, mean performance across all test questions, and cognitive demand across all test questions by test question levels and testing conditions.

		Test questions answered				Mean performance across all test questions				Cognitive demand across all test questions			
			
Groups by question level		*n*	*M*	*SD*	*d*	*n*	*M*	*SD*	*d*	*n*	*M*	*SD*	*d*
Overall	Adaptive	43	23.88	8.64	1.23^∗∗^	43	0.71	0.10	0.91^∗∗^	43	4.48	1.38	1.45^∗∗^
	Non-adaptive	49	15.47	4.88		49	0.60	0.14		49	6.48	1.41	
Level 1	Adaptive	43	12.00	5.57	2.39^∗∗^	43	0.77	0.09	1.14^∗∗^	43	5.40	1.63	0.90^∗∗^
	Non-adaptive	49	3.00	0.00		49	0.61	0.17		49	6.97	1.86	
Level 2	Adaptive	43	9.19	5.06	1.81^∗∗^	43	0.69	0.14	1.56^∗∗^	43	2.95	1.27	1.54^∗∗^
	Non-adaptive	49	3.00	0.00		49	0.46	0.15		49	4.81	1.18	
Level 3	Adaptive	43	2.44	3.48	0.22	24	0.67	0.20	0.17	24	5.42	2.38	0.87^∗^
	Non-adaptive	49	2.96	0.29		49	0.64	0.19		49	7.08	1.67	
Level 4	Adaptive	43	0.26	1.11	1.76^∗∗^	4	0.92	0.07	1.35^∗^	4	5.23	1.01	0.96
	Non-adaptive	49	6.47	4.77		46	0.68	0.18		46	7.18	2.13	


**n*, number of participants whose results were included in the analysis. ^∗^*p* < 0.05, ^∗∗^*p* < 0.001.*

Regarding performance on the test questions, we found that overall the adaptive group performed significantly better, *t*(90) = 4.30, *p* < 0.001, *d* = 0.91 (one-tailed: *p* < 0.001). This overall superiority was driven by the fact that the adaptive learners scored higher on Levels 1, 2, and 4 test questions, *t*(76.32) = 5.61, *p* < 0.001, *d* = 1.14 (one-tailed: *p* < 0.001), *t*(90) = 7.37, *p* < 0.001, *d* = 1.56 (one-tailed: *p* < 0.001), and *t*(48) = 2.53, *p* = 0.015, *d* = 1.35 (one-tailed: *p* = 0.008). There was no statistically significant difference regarding performance on Level 3 test questions, *t*(71) = 0.69, *p* = 0.495, *d* = 0.17 (one-tailed: *p* = 0.248).

Analyses of the cognitive demand of the test questions revealed that, on average, the non-adaptive testing group rated the test questions as more cognitively demanding than the adaptive testing group, *t*(90) = 6.87, *p* < 0.001, *d* = 1.45 (one-tailed: *p* < 0.001). This result was mainly due to the non-adaptive testing group reporting higher cognitive demands on Levels 1 and 2, *t*(90) = 4.27, *p* < 0.001, *d* = 0.90 (one-tailed: *p* < 0.001), and *t*(90) = 7.28, *p* < 0.001, *d* = 1.54 (one-tailed: *p* < 0.001). The non-adaptive learners also indicated higher cognitive demands on Levels 3 and 4, *t*(36.40) = 3.13, *p* = 0.003, *d* = 0.87 (one-tailed: *p* = 0.002), and *t*(48) = 1.80, *p* = 0.078, *d* = 0.96, (one-tailed: *p* = 0.039). However, due to the relatively low number of adaptive learners who reached Levels 3 and 4, these findings have to be interpreted cautiously.

For explorative purposes, we also analyzed the number of test questions that were answered in the learning phase. We found that the adaptive testing group answered significantly more test questions than the non-adaptive group, *t*(64.36) = 5.64, *p* < 0.001, *d* = 1.23 (one-tailed: *p* < 0.001). This effect was driven by the fact that the adaptive testing group received a statistically significant higher number of Levels 1 and 2 test questions, *t*(42.00) = 10.60, *p* < 0.001, *d* = 2.39 (one-tailed: *p* < 0.001) and *t*(42.00) = 8.01, *p* < 0.001, *d* = 1.81 (one-tailed: *p* < 0.001). Regarding Level 3 test questions, there was no statistically significant difference between the groups, *t*(42.50) = 0.97, *p* = 0.337, *d* = 0.22 (one-tailed: *p* = 0.169). However, the non-adaptive group answered more Level 4 questions, *t*(53.93) = 8.86, *p* < 0.001, *d* = 1.76 (one-tailed: *p* < 0.001). In view of these large differences regarding the number of received test questions, we included this learning process measure as a further potential mediator in our analyses.

##### Mediation analyses

To test whether the reported significant differences regarding learning processes mediated the superiority of the adaptive testing group in terms of learning outcomes, we conducted mediation analyses in the second step of our analysis. This was done by calculating a simultaneous multiple mediation model in which the dependent variable (the posttest scores) was regressed on the independent variable condition as well as the potential mediators performance on the test questions, the cognitive demand of the test questions and the number of test questions that were answered in the learning phase. Pretest scores were included as a covariate. By using the SPSS macro PROCESS (see Hayes, 2013), we tested the potential mediation effects by calculating 95% bias-corrected bootstrap confidence intervals from 5,000 bootstrap samples. For the sake of brevity, we limited the mediation analyses to the posttest scores regarding the question levels that showed statistically significant differences between the groups.

With respect to performance on the Level 1 posttest questions, we found a statistically significant indirect effect via the performance on the Level 1 test questions (a × b = -0.069, LCI = -0.133, UCI = -0.016). The indirect effects via the number of test questions on Level 1 and the cognitive demand of the Level 1 test questions did not become statistically significant (a × b = 0.067, LCI = -0.005, UCI = 0.152 and a × b = 0.022, LCI = -0.011, UCI = 0.052). Hence, the superiority of the adaptive testing group regarding performance on the Level 1 posttest questions was mediated by their higher performance on the Level 1 test questions but not by the number or the cognitive demand of test questions. For the Level 2 posttest questions, we found statistically significant indirect effects via the performance on the Level 2 test questions and the cognitive demands of the Level 2 test questions (a × b = -0.051, LCI = -0.108, UCI = -0.004 and a × b = 0.045, LCI = 0.006, UCI = 0.100). We did not find a statistically significant indirect effect via the number of Level 2 test questions (a × b = 0.006, LCI = -0.050, UCI = 0.065). These analyses indicate that the superior performance of the learners in the adaptive testing group on the Level 2 posttest questions was mediated via their higher performance on the Level 2 test questions as well as the lower cognitive demand they experienced while answering them.

### Testing vs. Note-Taking

In research question 3, we were interested whether testing would yield superior learning results than both note-taking and focused note-taking. There were no statistically significant differences between the two note-taking groups with respect to the overall score, *F*(1,92) = 0.02, *p* = 0.901, $\eta_{p}^{2}$ = 0.00 (one-tailed: *p* = 0.451), tested content, *F*(1,92) = 0.08, *p* = 0.777, $\eta_{p}^{2}$ = 0.00 (one-tailed: *p* = 0.389), and untested content, *F*(1,92) = 0.15, *p* = 0.702, $\eta_{p}^{2}$ = 0.00 (one-tailed: *p* = 0.351; see Table 1 for pretest and posttest scores for all four conditions). We therefore combined the two note-taking groups in the subsequent analyses.

To compare each of the two testing groups with the two note-taking groups, we used contrast analysis. Using contrast analysis is recommended by Wilkinson and Task Force on Statistical Inference (1999). For the analysis regarding the non-adaptive testing group, we assigned the contrast weights 0.5 for note-taking, 0.5 for focused note-taking and -1 for non-adaptive testing. The non-adaptive testing group did not outperform the note-taking groups on the overall score, *F*(1,140) = 0.14, *p* = 0.713, $\eta_{p}^{2}$ = 0.00 (one-tailed: *p* = 0.357), but only on tested content, *F*(1,140) = 14.55, *p* < 0.001, $\eta_{p}^{2}$ = 0.09 (one-tailed: *p* < 0.001). However, the note-taking groups showed better results than the non-adaptive testing group regarding untested content, *F*(1,140) = 16.25, *p* < 0.001, $\eta_{p}^{2}$ = 0.10 (one-tailed: *p* < .001). For the analysis regarding the adaptive testing group, we assigned the contrast weights 0.5 for note-taking, 0.5 for focused note-taking and -1 for adaptive testing. The adaptive testing group outperformed the note-taking groups on the overall score *F*(1,134) = 10.04, *p* = 0.002, $\eta_{p}^{2}$ = 0.06 (one-tailed: *p* = 0.001) as well as on tested content, *F*(1,134) = 53.68, *p* < 0.001, $\eta_{p}^{2}$ = 0.29 (one-tailed: *p* < 0.001), but showed a weaker performance than the note-taking groups on untested content, *F*(1,134) = 9.54, *p* = 0.002, $\eta_{p}^{2}$ = 0.05 (one-tailed: *p* = 0.001).

## Discussion

Our study contributes to the literature on the desirable difficulty of testing by casting light on two moderating aspects of the benefits of testing. Firstly, our results show that adaptation is a promising means to optimize specific testing. Increasing the fit between the learners’ current state of knowledge and the test questions increased the benefits of testing compared to non-adaptive testing. Therefore, the desirability of testing evidently can be improved by providing adaptive test questions. Secondly, our results indicate that non-adaptive specific testing leads to better learning of tested content than note-taking, even if the latter is supported by focus guides. These findings strengthen the case for implementing specific testing in real educational environments.

### The Benefits of Adaptive Testing

We found that adaptive testing yielded better results than non-adaptive testing for tested content and all of its subscales as well as for the overall score. Despite keeping learning time constant, participants in the adaptive testing group performed better on the posttest than the participants in the non-adaptive testing group. This was not only the case for familiar questions, but also for questions that the learners were not familiar with (i.e., near transfer and generation questions). Nearly all tested content effect sizes were of medium scale which puts the adaptation effects in the *zone of desired effects* within educational research (Hattie, 2009; *d* > 0.40 or $\eta_{p}^{2}$ > 0.039). The sole exception were the generation questions, which showed a small effect size. Regarding the two groups’ comparison with the note-taking control groups, the advantage of adaptive testing compared to non-adaptive testing becomes apparent, too: The very large testing effect for tested content found for the adaptive testing group ($\eta_{p}^{2}$ = 0.29) is distinctly bigger than the medium effect for the non-adaptive testing group ($\eta_{p}^{2}$ = 0.09), providing strong support for the implementation of adaptive testing. Mediation analyses suggested that this pattern of results was due in part to a higher testing performance in the learning phase on part of the learners in the adaptive testing group. This finding is in line with the notion that the extent to which learners can successfully respond to test questions is an important mediator of the benefits of testing (e.g., Rowland, 2014). Furthermore, we found a significant mediation effect via perceived cognitive demand. This result shows that the participants in the adaptive group outperformed the learners in the non-adaptive group not only because of their higher performance in the learning phase, but also because their mental resources were less exhausted due to the adaptation of the test questions to their level of knowledge. Hence, the learners in the adaptive group profited from the freed up capacity for the execution of beneficial learning processes (see Schnotz, 2010). Jointly, these findings reflect that the learners in the adaptive group actually received test questions that were better aligned with their current level of knowledge than the test questions provided to the learners in the non-adaptive group; this, in turn, was crucial for the benefits of adaptive testing to occur. Against this background, we conclude that adaptive testing can be more effective than non-adaptive testing.

However, it is important to highlight that this conclusive pattern of results was not found in terms of *all* learning outcome measures. First, the testing groups did not differ in terms of performance on the untested content questions. As both the adaptive and non-adaptive group did not have access to untested content items during their learning phase, this finding is not surprising. Second, regarding the tested content questions, no differences between the scores of the two testing groups on Levels 3 and 4 posttest questions could be found. At first glance, one explanation for this pattern of results could be that the adaptation mechanism caused the learners in the adaptive group to remain on Levels 1 and 2, where they answered significantly more test questions than the non-adaptive group. Due to limited learning time, this led to fewer learners reaching and answering the Levels 3 and 4 test questions. It would therefore be reasonable to attribute the results for Levels 3 and 4 posttest questions to the fact that many learners did not answer any Levels 3 or 4 test questions at all during the learning phase. However, our mediation analyses contradict this explanation. For performance on both the Levels 1 and 2 posttest questions, we found that not the number of test questions *per se*, but the learners’ performance on these test questions mediated the benefits of testing. This pattern of results suggests that the lack of superiority of the adaptive group over the non-adaptive group regarding performance on the Level 3 and Level 4 posttest questions should be due to a lack of significant differences between the testing groups in terms of their performance on both levels. However, this was only the case for Level 3 test questions. For the Level 4 test questions, we found a significant difference in favor of the adaptive learners. This surprising finding likely is due to the fact that only a small sample of participants (4 out of 43) of the adaptive testing group actually reached the Level 4 test questions in the learning phase, compared to nearly all participants (46 out of 49) in the non-adaptive testing group. Consequently, the superiority of the adaptive group regarding performance on Level 4 test questions should be interpreted very cautiously. Surprisingly, even though most of the learners in the adaptive group did not reach the Level 4 test questions (*n* = 19 learners did not even reach Level 3), they were not outperformed by the non-adaptive group regarding Levels 3 and 4 posttest performances. This suggests that the adaptive testing group had profited from working on the lower level test questions on Levels 1 and 2 to such an extent that it evened out the lack of practice on Levels 3 and 4.

Jointly, our results regarding the comparison of adaptive and non-adaptive testing are closely in line with the general notion that learning outcomes can be improved by matching the complexity of learning tasks to the learner’s current state of knowledge (see Kalyuga, 2007; Metcalfe, 2009). However, some important limitations apply. The first limitation concerns the non-adaptive testing condition. The test question sequence in our non-adaptive testing implementation followed a simple-to-complex-logic that was based on the notion that higher level questions are generally more effective than lower level questions (e.g., Jiang and Elen, 2011; Jensen et al., 2014; Roelle et al., 2018). Thus, the sequence was explicitly designed to relatively quickly provide learners with higher-level questions while also giving the learners the chance to practice retrieval on lower-level questions first. There are, of course, other possibilities to implement specific testing with questions of different complexity. One possibility would be a yoked control group design (e.g., Roelle et al., 2014; Kalyuga and Sweller, 2005) in which the non-adaptive learners receive the test question sequence of a matched counterpart in the adaptive group. Another possibility would be a design which poses the test questions in no particular order (Carpenter et al., 2008). Therefore, our results are limited to this specific set-up of testing. It remains to be seen how the adaptive approach employed in this study would fare against a differently designed non-adapted testing condition.

The second limitation concerns the cut-off values that were used to determine the sequence of the adaptive test questions. The cut-off values that were used led to the participants in the adaptive condition to remain on the Levels 1 and 2 test questions. Additionally, as mentioned above, only a few participants in this condition reached the Level 4 test questions. This raises the question whether the cut-off value used to advance to a higher level, namely 85%, was too high and blocked the participants from advancing to the higher-level questions. Although this might have been the case, it may have actually been advantageous to a certain extent. Grimaldi and Karpicke (2014) argue that it makes more sense to redundantly keep learners practicing the basics and give them more retrieval practice than to let them advance too quickly. This matches our finding that the non-adaptive group did not outperform the adaptive group on Levels 3 and 4.

Third, the adaptation mechanism as implemented in this study is not the only conceivable mechanism. It is reasonable to assume that learners who perform well on learning tasks such as test questions still might perceive themselves as being overloaded, which could affect their motivation and capacity to invest effort in subsequent (and more complex) learning tasks. In this case, adapting tasks not only to learners’ performance but also to their perceptions of the current cognitive demands could be beneficial. Therefore, more subjective measures such as the perceived difficulty of questions or the perceived cognitive demand could be a basis of adaptation that might be worth addressing in future research.

### Testing: Compared to What?

In previous research, testing has been mainly compared to restudy. However, due to the low educational utility of restudy, the true educational value of testing has been the subject of discussion in recent years. Our results show that the testing effect prevails against the presumably stronger control condition note-taking. This was also shown when note-taking was accompanied by focusing guides that aimed at canceling out any potential focusing advantages inherent to specific testing.

It should be highlighted, however, that the non-adaptive testing group did not outperform the note-taking groups on the overall posttest score. One explanation for this finding is that the note-taking groups had a clear advantage on untested content due to the way the experiment was set up, presumably canceling out the advantage on tested content by the non-adapted testing group. Only tested content was subject of both the testing and the note-taking conditions’ learning phase and thus only tested content enables a true comparison between testing and note-taking. Regarding tested content, the non-adaptive testing group clearly outperformed the note-taking groups.

The finding that the testing groups showed higher scores on tested content even though they performed worse on untested content than the note-taking groups indicates that although tested content and untested content was semantically related (see Note-Taking), acquiring knowledge of tested content did not require knowledge of untested content. It can therefore be assumed that the content which was designed as untested content was indeed not crucial for succeeding in understanding the tested content.

The results regarding untested content also provide evidence of note-taking’s utility as a learning method. After the initial learning phase, the participants in the testing conditions did not have access to the content that was the subject of the untested content questions. Therefore, the testing conditions equaled no-study conditions regarding the untested content. The two note-taking conditions performed substantially better on the untested content than each of the two testing conditions (medium effect). This underlines the assumption that note-taking is a suitable control condition (McDaniel et al., 2009; Rummer et al., 2017).

A further aspect that is worth highlighting is that our results regarding tested content contrast with those reported by Rummer et al. (2017), who compared note-taking to free recall (general testing). The outcome measure reported in their study is similar to our tested content because they did not include untested content in their experimental setup. In their first experiment, the testing effect did not show after 1 week and only appeared after a longer retention interval of 2 weeks, when they reported a medium-sized effect of *d* = 0.63 (or $\eta_{p}^{2}$ = 0.09). One possible explanation for the fact that in our study we did find a significant testing effect after 1 week is that feedback played a significant role. Unlike our study, Rummer et al. (2017) did not provide their testing condition with feedback. Their results could therefore reflect a common problem of test-based learning: Only that which is remembered can be learned. In our experimental setup, the learners received feedback that enabled them to identify their knowledge gaps and to better retrieve that information on subsequent questions.

It is also worth mentioning that, despite a randomized assignment of participants to conditions, the note-taking groups had higher pretest scores and therefore more prior knowledge than the testing groups. Although this is not ideal, the differences in prior knowledge do not delegitimize our results. Firstly, we included the pretest scores as covariates in our statistical analyses. Secondly, the note-taking groups having an advantage due to their higher prior knowledge would have hindered our hypothesized results, not helped them. Hence, our conclusion that test-based learning is more effective than note-taking in terms of fostering knowledge regarding tested content seems justifiable despite existing pretest differences.

As for the comparison of the two note-taking groups (with and without focus guides), it can be concluded that prompting learners to focus on specific content items did not have an effect on their learning outcomes. This calls into question the extent to which our implementation of the focus guides increased focus on the intended content. Our focus guides were visual markers that were designed to serve as a reminder for the learner to focus his/her attention on the relevant content. By contrast, the focus guides discussed in the literature, such as questions on targeted segments and elaborative investigation questions, are often designed to not only focus learners’ attention on certain content items but also elicit some kind of elaboration or retrieval (for an overview, see McCrudden and Schraw, 2007). However, as providing guiding questions would have changed the nature of the note-taking conditions, we decided to implement a mere visual form of guidance in the present study. One way to improve the effect of the focus guides could be to stress their intended function in the instructional text before the learning phase. This assumption remains tentative and should be tested in future studies.

Another limitation refers to the measures of the learning outcomes that were used. The posttest included questions that were similar (and partly identical) to the test questions, which warrants caution when comparing the learning outcomes of the testing and note-taking conditions. This may have led to an advantage for the testing conditions because they were already familiar with the question type. This could therefore partly explain the testing effect that was found regarding the tested content. However, the participants in the testing conditions also fared better on the generation questions (which were only used in the pre- and posttests) compared to the learners in the note-taking conditions. This shows that the testing effect found, albeit small, is not solely based on familiarity with the questions in the posttest.

### General Limitations and Future Research

In addition to specific limitations concerning the interpretation of the results regarding our research questions discussed above, attention should also be paid to two aspects of the experimental setup. First, it is important to highlight that our findings only apply to open-ended test questions. Research on testing suggests that close-ended questions such as multiple or single choice questions (e.g., Butler et al., 2007; McDaniel et al., 2011; Meyer and Logan, 2013) would have been possible alternatives; however, open-ended short-answer questions have been shown to be more effective than multiple choice questions (Kang et al., 2007). Combined with findings that show that higher-level test questions lead to better learning outcomes than lower-level test questions (Roelle et al., 2018), open-ended questions promised a higher chance at achieving the best learning results. On the other hand, multiple or single choice questions can be answered more quickly, which could be an advantage for adaptive testing because the sequence of test questions could be adapted more quickly. Adaptive testing with multiple or single choice questions would therefore be an interesting subject for future research.

Second, it is worth mentioning that in the present study we implemented testing in a closed-book format. That is, the learners were not provided with the opportunity to go back and watch parts of the e-lecture again while responding to the test questions. Even though a closed-book format is the usual format of testing, only very little research has shown it to be more effective than open-book testing (e.g., Blunt and Karpicke, 2014). Some studies have found that closed-book testing has either no or even detrimental effects in comparison to open-book testing (e.g., Agarwal et al., 2008; Roelle and Berthold, 2017). Hence, in addition to exploring (the refinements of) the adaptation mechanisms, a further promising path to optimize the benefits of (specific) testing could be to analyze the effects between closed- and open-book testing formats in future research and perhaps even design ways to combine these formats (e.g., first open-book then closed-book testing).

A third fruitful avenue for further research could be to investigate the effects of the type of testing (specific vs. general) in a more systematic manner. For instance, it can be assumed that specific testing and general testing lead to different mnemonic effects. Both specific and general testing rely on retrieval, but they may differ in terms of (the amount of) elaboration processes triggered by the learning task. During general testing, it is up to the learners how much elaboration takes place and which content items are covered; during specific testing, dependent on the type of specific test questions (e.g., lower-level retention vs. higher-level comprehension test questions), learners are forced to retrieve and elaborate on specific content items. Hence, in future studies it could be valuable to compare specific and general testing in terms of learning outcomes as well as learning processes. Regarding the latter, the number of covered content items and the degree of elaboration should be monitored. Moreover, in these studies it could also be fruitful to incorporate focused and unfocused note-taking conditions. Comparing both types of testing (specific/focused vs. general/unfocused) to both types of note-taking would provide deeper insight into the degree to which specific testing is desirable because it focuses learners on the relevant content items or engages learners in retrieval practice and elaboration.

## Data Availability Statement

The raw data supporting the conclusions of this manuscript will be made available by the authors, without undue reservation, to any qualified researcher.

## Author Contributions

SH, AG, KB, SF, and JR designed the experiments. SH organized the data collection and the data base. SH and JR performed the statistical analyses. SH, AG, SF, and JR interpreted the results. SH and JR wrote the first draft of the manuscript. AG, KB, and SF revised sections of the manuscript. All authors read and approved the submitted version.

## Conflict of Interest Statement

The authors declare that the research was conducted in the absence of any commercial or financial relationships that could be construed as a potential conflict of interest.

## Footnotes

1 In addition to the described measures, we also assessed a variety of other cognitive, emotional, and motivational measures. However, as these measures do not hold any relevance regarding our hypotheses, we do not refer to them in this manuscript.
